# Use of theatresports to promote positive education among youth participants

**DOI:** 10.1186/s12889-023-15825-2

**Published:** 2023-05-19

**Authors:** Gloria Hongyee Chan, T. Wing Lo, Johnny S. C. Fung

**Affiliations:** 1 Department of Social and Behavioral Sciences, City University of Hong Kong, Kowloon Tong, Hong Kong; 2 Research and Technology, Caritas Institute of Higher Education, Tsueng Kwan O, Hong Kong

**Keywords:** Applied theatre, Theatresports, Improvisation, Positive education, Youth

## Abstract

**Background:**

Due to the scarcity of research on the benefits of theatresports for youth, this study examined the outcomes of theatresports as a means to implement positive education in youth work settings.

**Methods:**

To this end, qualitative research was conducted with 92 participants in a theatresports program. Thematic analysis was applied to analyze the participants’ experiences of the program, using the framework of positive education.

**Results:**

Results showed that the processes and practices of the theatresports program helped the participants achieved well-being in terms of various domains namely positive emotions, positive health, positive relationships, positive engagement, positive accomplishment, and positive meaning. These capabilities and qualities acquired helped them achieve well-being, and the learning acquired from the program could even be applied to daily life situations and deal with the challenges.

**Conclusions:**

This shows that the theatresports program manifests the benefits of positive education. Corresponding implications were discussed.

## Introduction

Theatresports, developed by Keith Johnstone [1], is a form of theatre which involves teams of improvisers competing against each other in scenes and events on stage [2]. It enables improvisers to develop self-confidence and a range of interpersonal and intrapersonal abilities and qualities. These may include “creative thinking,” “public speaking,” “storytelling and communication,” “co-operation and team-building,” the mindset of “accepting mistakes and failure as a healthy component of a learning process,” “taking risks” [2], and greater exploration. These traits are consistent with features of positive education, which seeks to cultivate an all-round sense of well-being in emotional, psychological, and social dimensions [3–5]. This form of education is beneficial for students, particularly in Chinese contexts where the education system is famously competitive and puts significant emphasis on academic achievement [6], potentially resulting in mental health issues [7]. Owing to a lack of research specifically investigating the benefits of theatresports in youth settings, this study investigated the outcomes of implementing positive education with the use of theatresports. It is expected that practical implications for the future direction of youth work and youth education can be generated.

## Literature review

### Conceptualizing theatresports

Theatresports, characterized by competitions in improvisational games, does not focus on the "intent on winning" [1]. Instead, its essence lies in the improvisers’ “mutual desire to create dynamic, interesting theatre through spontaneity skills, storytelling and supportive play” [1]. It gives improvisers the courage to take risks and make choices based on positive human nature when being on stage, and it enables them to cultivate the ability to communicate with others using the self in terms of one’s senses and character [1, 8]. Also, it seeks to engage the audience in an atmosphere resembling sports events [1]. In theatresports, it is posited that good improvisers should understand what other people want [9] and build useful connections with the partners on stage, meaning that they can think inside the box (i.e., “being average”) [10, 11]. In this sense, the focus is on connecting with teammates and opponents to co-produce a good performance [1, 2].

Theatresports helps nurture not only performers’ various skills and qualities, such as storytelling, communication, team building, creativity, and self-confidence, but also a positive attitude and personal growth [2]. First, since improvisation is full of unpredictable events, improvisers are strongly encouraged to free themselves “from their cages” and take risks; indeed, risk-taking is viewed as a means of welcoming adventure into life, with failure being a normal part of this process [2, 12]. In this sense, failure is perceived positively as it entails learning by doing [11], rather than inability, problems, and mistakes. Second, the use of the self and personality in improvisation is emphasized over acting skills [8]. The authentic self or “being average” is highlighted [10], because attempting to be perfect adds to performers’ stress and fright [2]. Even “screwing up” on stage can prompt the audience’s support and affiliation if the performers “fail gracefully” [2, 12]. Third, during the dynamic story-building process with teammates and opposing teams, performers can nurture their openness, tolerance, multi-perspective thinking, and empathetic understanding of issues [13]. Furthermore, the narrative nature of improvisation makes it possible to reflect on and reconstruct life experiences, “which can facilitate contending with problems, questions, and uncertainty or surprises and unexpected results” [14].

### The benefits of theatresports for youth: in terms of positive education

Positive education is a form of education based on positive psychology [15], which is defined as “the study of the conditions and processes that contribute to the flourishing or optimal functioning of people, groups, and institutions” [16]. As a strengths-based practice [17], it is a form of education not merely for students’ academic achievement [18] but also “for both traditional skills and for happiness” [19]. Despite the differing models and approaches to positive education, the key elements of well-being to be nurtured among students to help them flourish can be summarized as: 1) positive emotions (“hedonic feelings of happiness”); 2) positive health (physical health and mental health); 3) positive engagement (“psychological connection to activities”); 4) positive relationships (“feeling socially integrated” and receiving social support from other people); 5) positive accomplishment (“a sense of achievement” and “feeling capable”); and 6) positive meaning/purpose (“believing that one’s life is valuable and feeling connected to something greater than oneself”; being “spiritual, humanitarian, or otherwise altruistic”; “contributing to others and the community”) [4, 20–23]. Flourishing means “feeling good” (“hedonic…experiences such as feeling content” and “hopeful about the future”) and “doing good” (the “eudaimonic approach”, which “focuses on equipping students with the skills and knowledge that help them to thrive when faced with both challenges and opportunities”) [3, 4]. In sum, positive education is a form of education that offers opportunities for students to cultivate their character strengths (“individual positive traits that are widely valued”) and capabilities, including empathy, social skills, emotional competencies, problem-solving skills, resilience skills, and a growth mindset (“talent and intelligence are seen as malleable and can be developed further with effort and persistence”), so that they can function and cope with challenges adaptively [4, 20].

When theatresports is applied in youth settings as an educational approach, it can be implemented as a tool for positive education, in two key ways. First, given that theatresports is characterized by “spontaneity” [2] and “immediacy” [1], sources and materials for storytelling and improvising typically come from daily life events [2]. Spectators can be genuinely engaged by the “lives” and “experiences” of the improvisers and the audience alike [2]; in this way, thinking inside the box and “being average” [10, 11] is more important than being selfish and striving for personal excellence. As participants are required to play theatresports with the use of self, they inevitably undergo constant self-reflection and build positive character, such as having a “good nature” [2], being benevolent [2], overcoming an aggressive urge to win, being happy to accept challenges, and embracing failures. Furthermore, positive education is usually implemented through teaching certain skills via specific activities [4, 20]. When using theatresports as a tool for positive education, the education process will become much more interactive. This is because theatresports is “a live art” characterized by “the simultaneous presence of performers and audience” and “immediacy,” typified by constant communication and interaction among improvisers as well as between improvisers and the audience [1]. Involving these types of interaction in the process of positive education, participants will be able to achieve all-round well-being at three levels—micro (personal), meso (social), and macro (community) [24]. Whilst positive education largely emphasizes personal well-being (e.g., hedonic happiness), the nature of theatresports which nurtures all-round well-being (especially benevolence, collaboration, and relationship building) makes positive education more applicable to Chinese cultures, where social relationships and social well-being are highly valued [25–28].

**Fig. 1:**
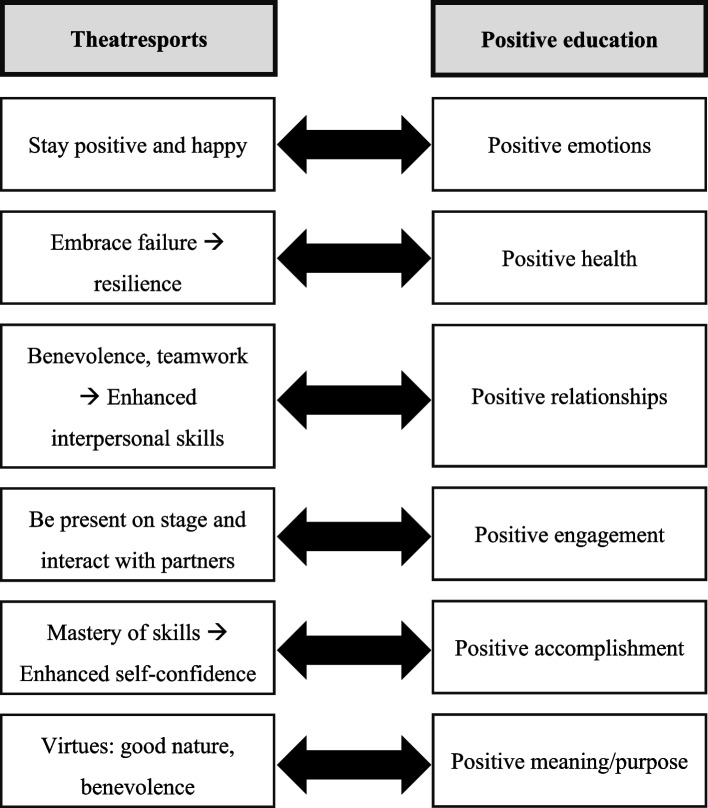
How theatresports manifests the features of positive education

Theatresports is consistent with the aim of positive education, which is to cultivate students’ general well-being [3–5]. As annotated in Fig. 1, the features of theatresports can match with the six key elements of well-being in positive education. Through upholding the philosophy of taking risks and embracing failures as a learning process [2], participants are encouraged to “stay happy” with the mistakes they make [2] (i.e., positive emotions). Thus, improvisers are encouraged to “retain a positive state of mind and deal with failure or loss” [2], which is related to the concept of resilience, defined as the ability to overcome adversities and achieve normal functioning [29] (i.e., positive health). In theatresports, the priorities are not to perform to the best of one’s abilities or to defeat one’s opponents, but rather to be good-natured and display benevolence and teamwork with partners on stage [2]. Theatresports also helps nurture improvisers’ interpersonal skills [2] (i.e., positive relationships). During improvisation, it is essential to be “present” on stage to notice and react to what’s happening and accept teammates’ ideas [2] (i.e., positive engagement). Through mastering communication and storytelling techniques, improvisers are able to increase their self-confidence. Since theatresports values amiability and benevolence, improvisers are given the opportunity to nurture their inner self through their continued development of their authentic self (i.e., positive meaning/purpose) (see Fig. 1).

### Current study

**Fig. 2:**
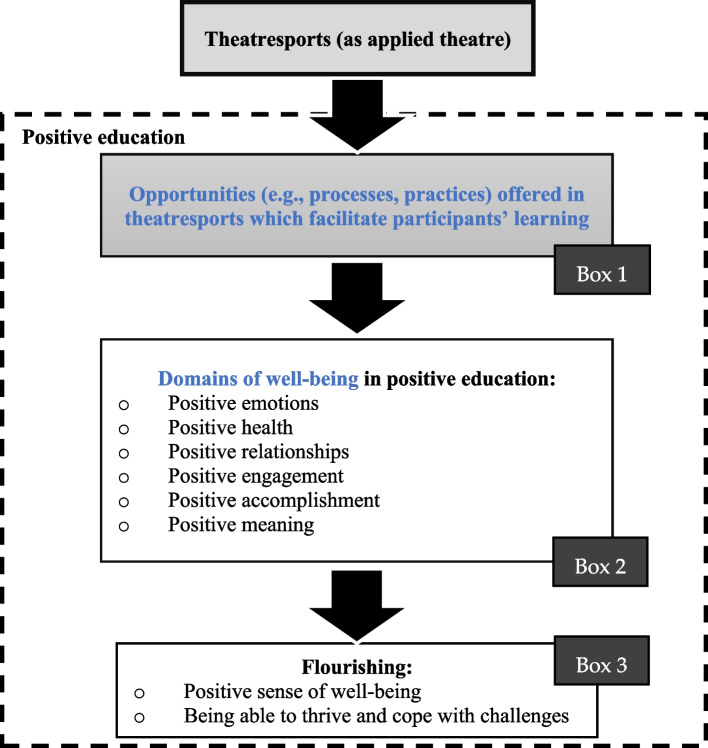
Theoretical framework: The application of the elements of positive education into theatresports

To summarize, positive education is a kind of strength-based education which nurtures the well-being of individuals through "conditions and process" [16]. The above literature demonstrates how theatresports manifests features and benefits of positive education. As annotated in Fig. 2, using theatresports to implement positive education involves the opportunities (e.g., processes, practices) offered in the theatresports program which have learning values (enhancement of skills/abilities) (Box 1), so as to nurture the participants' well-being. Participants' well-being can be nurtured through six key elements namely "positive emotions", "positive health", "positive relationships", "positive engagement", "positive accomplishment", and "positive meaning" [4, 20–23] (Box 2). Through these six domains of well-being, flourishing can be achieved, in which one becomes able to deal with the challenges adaptively (Box 3). In this study, which aimed at investigating the outcomes of the theatresports program, the participants’ experiences were analyzed based on this theoretical framework (see Fig. 2). Through this study, it is expected to provide valuable implications regarding the future direction of youth education.

## Methods

### Participants

A total of 92 individuals took part in this study, all of whom were participants of a theatresports program implemented by a youth service agency in Hong Kong. The research was approved by the Research Ethics Committee of City University of Hong Kong. All methods were carried out in accordance with procedures and guidelines of Human Subjects Ethics Sub-Committee of City University of Hong Kong and the Social Workers Registration Board. The program is characterized by: 1) 18-h training workshops, which are taught by social workers who have received prior theatresports training. The workshops help participants learn the rules of the games as well as the related performance techniques. The social workers are supported by professional theatre practitioners and tutors/assistant coaches (senior, more experienced participants who provide guidance for the junior ones) in the theatresports program; 2) regular weekly practices for the theatresports games, which involve different scenarios for the participants to apply what they have learned from the training workshops; and 3) theatresports competitions, which are held for the participants to further consolidate their learning. Participants of the program have to meet the attendance requirement of 85% in the workshops and the practices in order to join the competitions. The researcher collaborated with the agency to evaluate the outcomes of the theatresports program. In order to gain a greater understanding of the program, the researcher also attended the theatresports training workshops and competitions before the research period.

Under the research collaboration with the agency, participants were recruited by the agency, then passed to the researcher for subsequent data collection. Informed consent was obtained from all participants or their parents prior to the study. Of the total sample, 61 (66.3%) of them were male and the rest (*N* = 31, 33.7%) were female. The participants’ age ranged from 12 to 27 years, in which 56.5% (*N* = 52) of them were aged between 14 and 17. Regarding education level, the majority of the participants were Secondary 1 to Secondary 6 students (equivalent to Year 7 to Year 12 students), whilst the oldest ones were attaining or had attained tertiary level of education. Their length of participation in the program ranged from 2 months (*N* = 10, 10.9%) to 4.5 years (*N* = 7, 7.6%); more than half of the participants (*N* = 53, 57.6%) had joined the program for 6 months to 1 year. Due to the age range of eligible youth service targets of 12–29 [30], participants of this program cover heterogenous age groups.

The participants join the program differently based on their age groups. For those studying Secondary 1 to 3, they participate in the training workshops in their schools. For those who have completed Secondary 3, they can choose to stay in the program and continue their participation as service users of the agency. Additionally, for participants who do not attend school, they are recruited through outreach (e.g., in parks) by social workers. Among the 92 participants, 55 of them (59.8%) were recruited through schools, whilst the rest of them (*N* = 37, 40.2%) were recruited through outreach. Despite the differences in how they participate in the workshops, they join the competitions together, regardless of age differences. Moreover, following the 2-month training, the participants can stay in the program and continue practicing the games and joining the theatresports competitions. Some senior participants even continue their participation in the program through becoming tutors/assistant coaches.

### Data collection procedure

To understand their perceptions of the program, participants were invited to share their thoughts and insights during a two-day theatresports competition held by the agency. The 92 participants joined either the first or the second day of the competition. The sharing sessions were held at the end of each day. Before the sharing sessions, debriefing sessions were held by the social workers to help the participants share their thoughts and feelings, and to review and consolidate their experiences. After that, the sharing sessions were held, in which the participants were divided and paired with 51 research team student helpers. The student helpers were Master students, studying Final Year of a counseling program of a university in Hong Kong. These students were invited to join the research team due to their knowledge and abilities of undergoing interactions with youth, which made them suitable for conducting unstructured interviews with the participants in this study. Before the study, all the student helpers attended a briefing session to understand how the sharing sessions would be delivered to collect the responses from the participants.

During the sharing sessions, each participant was asked the following open-ended question: "Please share the most important thing you have learned since joining the theatresports program, giving examples.", and share their thoughts and feelings with no time limit. The sharing sessions were conducted in Cantonese, in the form of unstructured interviews. An open-ended question was asked as a means of data collection, because it allowed the participants to narrate rich, in-depth stories which captured their experiences, changes, and growth from their perspectives. Their sharing was not limited to the theoretical concepts of positive education. Rather, the researcher coded and analyzed the participants’ narratives using the theoretical framework of positive education, to investigate if the program did manifest the characteristics and outcomes of positive education. The sharing was audio-recorded. The average duration of each individual sharing was 18 min.

### Analyses

Thematic analysis was performed to analyze the verbatim accounts of the participants, adhering to the following steps: 1) “familiarizing with the data”; 2) “generating initial codes”; 3) “searching for themes”; 4) “reviewing themes”; 5) “defining and naming themes”; and 6) “producing the report” [31]. First, the audio-recorded accounts of the participants were transcribed into written Chinese, followed by organizing the transcripts in an Excel spreadsheet and studying them. Then, the researcher generated codes from the data, by associating meaningful data to generate themes and subthemes for analysis. Based on existing literature on positive education, themes and subthemes were developed in a theoretically driven manner [31]. Aligned with the theoretical framework (Fig. 2), the themes included: 1) the processes and practices contributive to participants’ learning of capabilities (i.e., Boxes 1 of Fig. 2); 2) domains of well-being in terms of positive emotions, health, relationships, engagement, accomplishment, and meaning (i.e., Box 2 of Fig. 2); and 3) achievement of well-being/flourishing (i.e., Box 3 of Fig. 2). Besides, based on the unstructured, narrative nature of the qualitative data collection, participants had expressed feelings other than the proposed themes. These feelings and experiences were also analyzed to enrich the results. After the establishment of themes and sub-themes, these themes, sub-themes, and the relevant verbatim accounts were translated into English by a specialist. The coding of the transcripts was performed by a researcher, then reviewed by two other researchers of the research team and cross-checked by six social workers of the agency. In case there are discrepancies, research team members underwent discussions to resolve the inconsistencies. The analyzed data was also cross-checked by the youth service directors working on the program.

Table 1 summarizes the coding of the qualitative data, with the number of participants whose responses fell within or were related to each sub-theme.

**Table 1:** Themes in the qualitative results

Themes	Subthemes	Examples of codes
The processes and practices contributive to participants’ learning of capabilities	Theatresports games (*N* = 8)	N.A
	Input of the practitioners (tutors/assistant coaches, social workers) (*N* = 7)	Teaching, guidance, spiritual support, company
Nurturing flourishing through the six domains of well-being	Positive emotions (*N* = 12)	Happy, hope/hopeful, positive
	Positive health (*N* = 3)	Perseverance, resilience
	Positive relationships - Acquisition of interpersonal skills which helped the participants build positive relationships with others ▪ enhanced speaking skills (*N* = 5) ▪ enhanced social skills (*N* = 21) ▪ understand the importance of teamwork ◆ enhanced team spirit (*N* = 41) ◆ enhanced sportsmanship (*N* = 18) - The positive relationships established among the participants serving as peer support for the participants ▪ friendships with other participants were valued (*N* = 18)	Communication skills, collaborative skills, sense of team spirit, friendship, support from peers
	Positive engagement - Engaging in the program and perceiving it as interesting (*N* = 12) - Involvement in the program, dealing with challenges in theatresports (*N* = 15), and make breakthroughs (*N* = 16) - Skills/capabilities developed: ▪ observational ability (*N* = 1) ▪ enhanced creativity/imagination (*N* = 3) ▪ problem-solving skills (*N* = 5) ▪ multi-perspective thinking (*N* = 4) ▪ undergoing self-reflection (*N* = 2) ▪ achieving self-understanding (*N* = 5)	Involvement in activities and make breakthroughs, thus developed capabilities
	Positive accomplishment - engaged the interests of participants who were interested in acting/performing (*N* = 8) - being able to showcase self (*N* = 12)	Actualization of own interests and potential in acting/performing; I am “someone”
	Positive meaning (*N* = 9)	Making contributions, gaining meaning in life
Achievement of flourishing	Inner sense of well-being -self-confidence (*N* = 25)	Self-confidence
	Applying the learning from the program to cope with challenges in life (*N* = 3)	Application of learning (e.g., skills and competencies) into daily life to deal with issues and problems
Other perceptions and feelings	Negative emotions (*N* = 5)	Negative emotions from losing competitions and feeling disappointed

## Results

### The processes and practices contributive to participants’ learning of capabilities

The staff implementing positive education; the teaching and learning of concepts; the application of abilities, values, and mindsets; and the engagement in reflection can all be regarded as processes of positive education that help participants achieve well-being and flourish [5]. We found that not only the theatresports activities themselves but also the input of the practitioners helped participants understand theatresports’ values and acquire various performance and interpersonal/collaborative/team-building skills.

#### Theatresports games

Some participants expressed that the theatresports games had learning values (*N* = 8). Participant P33 gave a detailed account of this point: *“People may only see games or improvisation. But they all involve techniques. Interaction is fundamental…when we don’t accept the offers from the partners/opponents, we are blocking them and the story cannot develop*.” This showed that playing the games could enhance the participants’ skills for producing a good performance for the audience.

#### Practitioners (Tutors/assistant coaches, social workers)

Some participants mentioned that they had learned from their tutors/assistant coaches (*N* = 6). P19 described the role and significance of the tutors/assistant coaches, who were originally responsible for teaching the games: *“[D]uring the process, the tutor not only leads us and teaches us how to collaborate with teammates, communicate with people from different circles, listen to them, and accept their opinions, but also, most importantly, helps us understand that we need to respect the results and every participant.”* Similarly, P84 expressed that *“the tutors/coaches encourage the participants to learn and try.… I didn’t imagine that I would be able to do it!”* In addition, P25 was grateful for their social worker’s care, spiritual support, and company: *“During the process, we were not united at all. However, the social worker did not blame us…. Every time we finished our practice, he gave us comments and encouragement.”* To summarize, the guidance and support from the assistant coaches and the social workers helped the participants acquire intrapersonal capabilities (e.g., positive attitude) and interpersonal capabilities (e.g., social skills).

### Achieving flourishing through different domains of well-being

#### Positive emotions

Positive emotions pertain to individuals’ emotional experiences (e.g., feelings of “joy,” “hope,” and “gratitude”) and their “capacities to anticipate, initiate, experience, prolong, and build positive emotional experiences” [4]. In other words, this domain of well-being not only refers to the “maximization of pleasure and the minimization of pain” but also their adaption of “healthy responses to negative emotions” [4]. Applying such domain of well-being into theatresports, it refers to positive emotional experiences of the participants as well as the nurturance of their positive thinking which constitute their capacity to deal with negative emotions. Given that there were no absolute definitions of right or wrong in improvisation/theatresports, the participants developed positive thinking (*N* = 12). P70 expressed that *“maybe we don’t have good acting skills, but theatresports emphasizes positive and hopeful messages. We used to think in negative directions; now we’ve gradually become more positive.”* P1 also expressed that *“I gained a lot of positive energy [from the program]; even when I face stress, I don’t think it is something very important.”* This showed that by allowing alternatives and possibilities, theatresports helped participants understand that there were always hope and adaptive ways to deal with problems and adversities, which were related to psychological well-being [4]. As a result, the participants remained joyful when participating in the program. Participants expressed that they were happy when participating in the program (*N* = 13). As said by P66, “…I felt so happy. I enjoyed the whole process very much”. Their happiness reflects that they have endorsed the values and philosophy of theatresports.

#### Positive health

Although the program’s benefits for physical health were not made prominent by the participants, certain advantages in terms of psychological or mental well-being were noted. According to P13, theatresports required problem-solving. P49 recalled his experience in a competition: *“My teammate was on stage, but he suddenly couldn’t continue and it seemed like the opposing team was going to win. What I could do at that time was to go out and help him cope with this problem. Finally, we overcame it and we were strong enough to remain in the competition. We did not give up.”* Similarly, P48 expressed that *“when we face difficulties in our daily lives, it is easy for us to back off and give up, but when we are on stage and cannot get away, we have to face that challenge at that moment.”* This showed that the participants had learned to persevere and be resilient when encountering issues during the competition. P42 even claimed to embrace failures: *“I value successes and achievements less.… [H]aving experienced failures, [I’ll] make improvements,”* showing a positive attitude when experiencing challenges and adversity.

#### Positive relationships

The building of positive relationships with others requires the acquisition of social and emotional competencies [4]. Many of the participants claimed to have acquired various interpersonal skills and viewed these as useful:

##### Acquisition of interpersonal skills which helped the participants build positive relationships with others

To begin with, a few participants noted that theatresports had nurtured their speaking skills (*N* = 5). For instance, P3 shared that *“since joining this program, I no longer stutter and I am able to speak more fluently.”* As a form of theatre that required immediate, spontaneous interactions among improvisers, theatresports encouraged participants to refine their communication skills to improve their performance. Furthermore, many of the participants claimed to have enhanced their social skills (*N* = 21). For example, P41 shared that *“as we had to prepare for the competition, we had more time to meet up…. We had more opportunities to learn how to express our own thoughts and listen to other teammates’ thoughts. We had more interactions than before.”* P10 expressed that theatresports *“enhances my ability to work with other people,”* while P22 echoed this, saying, *“I used to be a selfish, timid and bad-tempered person who tended to care little about other people’s feelings. But when we are on stage, we cannot show our emotions recklessly. Hence, I’ve learned to control my emotions and learned when to speak and act appropriately.”* In addition, some participants expressed that they had learned to handle conflicts adaptively. For instance, P76 shared that *“although we’ve experienced many conflicts and had different opinions, we will share [our feelings] with one another and learn to respect one another, so as to come up with a mutually agreed decision.”* This showed that the program helped participants enhance various social skills, such as effective communication skills, empathy, and active listening skills, and these skills facilitated better collaboration with teammates and maintenance of good relationships with others.

Apart from the above, many participants expressed that they came to understand that teamwork was important in theatresports, and it helped them nurture a team spirit (*N* = 41). As P67 stated, *“Theatresports is not something that can be accomplished by just one person; it requires many people to be involved in it and to work well with one another.”* The participants realized that it was more important and valuable for each member to make the best use of their strengths and to perform their roles as a team than to strive for their own personal honor. As P86 noted, *“the whole team would feel satisfied and happy with the joint effort made by every member.”* The participants understood that the essence of good team spirit included friendly relationships among teammates (P57), communication among teammates (P81), trust among teammates (P50), mutual respect for one another’s ideas (P62), mutual dependence and collaboration (P31), accommodating one another (P14), and camaraderie (P19). It is noted that despite the competition against the opposing teams, the participants recognized that sportsmanship was essential (*N* = 18). They did not act aggressively towards them; instead, they thought that the process of the competition, finding enjoyment and areas for further self-enhancement, were more important than winning (P2, P4, P13, P42, and P55). They developed good relationships not only with their teammates but also with opposing teams (P65). They understood that the essence of theatresports was not to aggressively compete for one’s own fame and self-interest; rather, the competing teams shared the same goal of producing a good show together on stage (P24). As P88 said, *“The two competing teams are not really in a hostile relationship. Instead, the teams can mutually help each other and solve problems together, so that both teams can survive the competition. The two teams are actually mutually beneficial.”* This demonstrated that the participants realized the true meaning of sportsmanship in improvisation [2, 11, 12].

##### The positive relationships serving as peer support for the participants

The positive relationships established among the participants in the program serve as an important source of social support for the participants. Participants recognized that the program provided opportunities for them to meet friends and form friendships with other participants (*N* = 18). For instance, P89 stated that *“the program expanded my social circle,”* while P21 noted that the program *“helps me meet different people. I have no friends at school and tend to nestle away deep in myself, but in the program I can open myself up.”* Many valued peer support (*N* = 39) as an important aspect of the program:

*Since joining theatresports, I have laughed, played, and cried with my peers.… I feel the warmth…. Since then, I have become more optimistic and even more hyper…gradually, my suicidal thoughts have faded away.* (P26)

*As we’re all extroverts, we feel relaxed when we play with one another. We understand each other’s thoughts. In addition to theatresports activities, we play outside together. We’ve become good, intimate friends.* (P31)

Peers helped the participants experience the power of collective effort in handling issues, and offered them great emotional support to soothe their negative emotions. They also found a sense of belonging to the theatresports family. The friendships gained from the program even extended to their daily lives, offering them continued support.

#### Positive engagement

Strong engagement can be regarded as the concept of flow, which is “a state of intense absorption and optimal experience that results from taking part in intrinsically motivating challenges” [4, 32]. Participants’ verbatim accounts showed that they were highly engaged in the program; not only did they perceive it as interesting, they were also involved in the processes in the program and deal with the events that occurred. These were illustrated as below:

##### Perceiving the program as engaging and interesting

As a program characterized by learning distinctive games and competitions, some participants expressed that the process was interesting (*N* = 4). For example, P41 appreciated the form of the competition, deeming it fresh and fun. This was the reason why P34 stayed in the program: *“I insist [on joining the theatresports program] because it is interesting and funny.”*

##### Involvement in the program and dealing with challenges in theatresports

The participants showed significant involvement and engagement in the events that occurred in theatresports. Several claimed that theatresports required a degree of proficiency to adapt to changes (*N* = 15). As P72 said, *“As many things [lines during storytelling and improvising] are instant and improvised on stage, you need to think fast and react fast in order to play the games.”* Furthermore, P61 noted that *“there is not much time for preparation, so it is important to have the ability to adapt to changes and give responses instantly, as well as to have creativity.”* Given that improvisers experienced everything on stage and could not escape (P48), some participants expressed that the program had encouraged them to step out of their comfort zone (*N* = 16). They realized the potential for all sorts of challenges to occur on stage, forcing them to break through their limitations. As P82 said, *“The most challenging aspect is that I need to do things with which I’m not familiar…like games which require speaking.”* As such, the participants in the program were encouraged to make breakthroughs, overcome their fear of failure courageously, and make new attempts and learn, so as to achieve continuous self-enhancement.

##### Capabilities and qualities developed as a result of engagement in theatresports

As a result of coping with theatresports’ unpredictability, certain participants mentioned that they had improved their: 1) observation skills (*N* = 1) (P32: *“Very often, we need to give spontaneous responses to other improvisers. Whether we can respond appropriately depends on how much we have learned from our daily lives. Improvisation…nurtures my observational ability.”*); 2) creativity (*N* = 3) (P56: *“From this competition, I’ve learned many things; my skills for expression and my imaginative ability have been enhanced”*); 3) problem-solving skills (*N* = 5) (P13: *“Every game requires problem-solving ability. Every time we learn [the game] or are in competitions, we face a lot of situations that require us to solve problems…together with our teammates”*; P90: *“When we’re outside, facing other people, I’ve understood that we need to handle emergencies adaptively, such as thinking of ways to minimize dead air moments”*); and 4) multi-dimensional thinking (*N* = 4) (P53: *“We…have to learn to think from different perspectives in order to generate more solutions [when improvising on stage]”*), so as to improvise lines and develop meaningful stories on stage. Besides, engaging in theatresports activities encouraged self-reflection of the participants (*N* = 2). For instance, P10 expressed that *“I engaged in deep self-reflection after the previous competition. Because it was my first competition, we were very nervous and invested a lot of effort in the opening. We hadn’t practiced the games much.”* This suggests that participants reviewed and evaluated the process of theatresports and found ways to improve, using their “intrinsic motivation” [4]. As such, participants became increasingly aware of their abilities [4]. Several participants expressed that theatresports had fostered their self-understanding (*N* = 5). For example, P84 said, *“From theatresports, I understand my weaknesses and limitations.”*

#### Positive accomplishment

Positive accomplishment involves “the development of individual potential” [5]. Some participants believed that the program engaged the interests of participants who were interested in acting/performing (*N* = 8). Some participants also mentioned that the program provided a stage on which they could showcase themselves (*N* = 12). For example, P25 expressed that *“I want to sing and I want to perform on stage, being recognized as ‘cool’ by the others. I wish to see myself as ‘cool’ too.”* P40, too, expressed how *“during the process of joining theatresports competitions, I see my value.”* This showed that the program helped the participants recognize themselves as “someone.”

#### Positive meaning

Making contributions for others and viewing one’s life as valuable and purposeful contributes to psychological well-being [4, 33]. Several participants expressed how they had found meaning in life (*N* = 9). For instance, P11 expressed that *“Theatresports is a meaningful activity. Although there are no special gifts or monetary awards, there are other life-related insights”*. During the program, P91 even contributed by teaching junior participants what he had learned from the stage: *“Consequently, my values and attitude about life have changed. Since becoming a tutor, I have the responsibility to teach other people what I have learned. This gives me a new meaning in life.”* Such fruitful and meaningful experiences became unforgettable for the participants.

### Participants’ well-being

Well-being refers both to feeling good and functioning well [21, 34]. For participants in the program, they achieved an inner sense of well-being through the aforementioned six domains of well-being. They also found these capabilities useful for dealing with the challenges in daily life.

#### Inner sense of well-being

A number of participants noted that their self-confidence had increased (*N* = 25) since joining the program. For example, P1 shared that *“I used to be very shy, for fear that people would see my negative side…[but] after taking part in theatresports groups and activities, I’ve become more confident…. I no longer fear how other people see me.”* Likewise, P20 claimed that *“I used to be timid and feel inferior; as I’m short, very often I experience limitations when doing things. Theatre helps elevate my self-confidence.”* This shows that participants appeared to feel less vulnerable when confronted with challenges after joining the program.

#### Applying the learning from the program to cope with challenges in daily life

A few participants even mentioned that they perceived the competencies (e.g., interpersonal skills) they had learned from the program as applicable to solve problems related to interpersonal conflicts in daily life (P13, P67), and that newly developed character could sustain their daily lives (P16). For example, P16 said that *“since joining the program, I’ve become more positive in my daily life.”*

### Other perceptions and feelings

In spite of the significant positive feedback and outcomes generated by the program, a few participants expressed negative emotions and feelings, due to not being able to win the competitions:

*After taking part in numerous theatresports competitions, my deepest feeling is disappointment. This is because we’ve been practicing very hard in every competition, but we keep on losing each time. It’s frustrating.* (P75)

*I feel stressed during competitions; the pressure makes me barely able to speak at that time. In the end, we lost. I felt heartbroken.* (P79)

This reflects that although theatresports emphasizes processes (e.g., use of authenticity, instant interaction with others, sportsmanship) rather than outcomes (i.e., winning) [2, 8, 11], some participants appeared to be outcome-oriented, exhibiting a strong ambition to win instead of simply enjoying the experience. For instance, P79 expressed that *“in the end, we could not achieve the expected outcomes and could not get into the final, so I was disappointed. Winning in the competition is of the utmost importance, not friendship or happiness…. [I]f someone thinks that losing in a competition is not a big deal, then this person is not suitable to join theatresports.”*

In these cases, the sense of disappointment for P75 might indicate a lack of successful experience that would otherwise have engendered a sense of achievement, while for P79, his feelings of stress and despair suggested that success was linked to self-worth, and thus he needed to increase his resilience in response to stress. Hence, further support was required to help participants handle distress and despair related to the competitions, and to review the meanings of “success” and “loss,” [2, 11, 12], so that they could gain insights into their fear of failure and rethink how they should view this. Furthermore, P37 expressed a sense of emptiness following the competition: *“Actually I feel a bit unhappy, because I did not achieve good results to repay my teachers and tutors…also, it seems to me that the goal has gone…. I don’t know what to do next…if I had the chance, I would like to take part in the competition again.”* Competitions were important as they provided opportunities for participants to develop a positive identity—of being “someone”—on stage. However, once the performance ended, feelings of loss emerged. Again, this reflects the necessity of further support to help participants deal with these feelings and to consolidate such experiences as valuable memories or assets that could enable personal growth.

## Discussion

The results showed that the theatresports program demonstrated the features and outcomes of positive education [3–5]. In terms of positive emotions, having endorsed the values of theatresports, the participants understood the value of embracing failures [2] and learned to stay positive and hopeful in order to cope with challenges. As such, the participants enjoyed the program and felt happy. In terms of positive health, given the spontaneity of theatresports [2, 12], events were unpredictable and participants had to cope with everything happening on the stage. Thus, the participants learned to deal with challenges adaptively and to embrace failure, enhancing their resilience. In terms of positive relationships, the participants acquired various social skills, especially the importance of teamwork, which helped them maintain good collaborative relationships with others, receive peer support, and even expand their social circle in real life. In terms of positive engagement, the participants engaged in the program out of interest. Due to the spontaneity and unpredictability in theatresports [2, 12], all the participants could do was to “be present” on stage [2], fully attend to the events happening there and “take risks” in response to any challenges [2]. Thus, during the process, they made breakthroughs, developed various capabilities, and also gained self-awareness of their strengths. In terms of positive accomplishment, given that the participants felt that they had actualized their capabilities and talents, they recognized their value. In terms of positive meaning, the participants perceived their experiences of theatresports as insightful and fruitful, reflecting that the program is more than just an activity of interest. Consequently, they achieved a sense of well-being in terms of their self-confidence, efficacy, and self-actualization. Furthermore, the character traits they developed through the program could be applied to handle challenges in their daily lives. All these outcomes suggest that theatresports has significant potential to be applied as a form of positive education.

Among the results, the facet of positive relationships appeared to be the most prominent. A considerable number of participants mentioned the significance of peer relationships. Peer groups are widely recognized as having the following functions: 1) bringing about social support for youth, particularly when they face problems in life [35], and thereby promoting their positive development [36]; 2) establishing a sense of identity, self-concept, and self-worth [36], relevant to Erikson’s [37] psychosocial stages, whereby adolescents search for a sense of self and identity via social interactions to prepare for the future; and 3) serving as an agent of socialization in terms of the development of personality and the learning of behavior, attitudes, knowledge, and skills [38, 39]. In Chinese contexts where the maintenance of social relationships is important [26], providing platforms that are capable of cultivating youth’s social skills and nurturing peer support for them will be particularly useful: not only do the platforms fulfill their developmental needs, but they also help them achieve a sense of well-being through the development of a social self.

On the other hand, several participants were aggressive or excessively determined to win, exhibited negative emotions when they failed, and appeared to be overinvolved in the competitions, potentially causing them to bypass the self-reflection aspect of the program. Such issues are contrary to the program’s intended outcomes and suggest that further support is probably needed in aspects such as team building, conflict resolution, and inner self-development. The outcomes of positive education are affected by education providers’ skills, attitudes, and experiences, their understanding of underlying theoretical concepts, and their intervention approaches [40, 41]. In order to better relate the essence of improvisation [2, 8] to participants’ self-development, practitioners and education providers must have a thorough understanding of the philosophy and concepts of theatresports and positive education, and to endorse their value, so as to enhance the delivery of the latter through the former. To help the participants consolidate their learning, they may, for instance, facilitate participants’ reflection through in-depth discussions and debriefing following the activities [42, 43].

## Conclusions

Theatre is an art form that not only meets the interests of many young people but also helps develop their self-growth, capabilities, and sense of well-being [44, 45]. The findings suggest that theatresports can be used to implement positive education to cultivate youth’s all-round well-being. To optimize the educational outcomes, however, practitioners or education providers should be afforded a more significant role than is currently the case in terms of team building and intrapersonal reflection, in order to maximize participants’ personal growth.

To date, research on theatresports is scarce. This study is significant in enriching the research in terms of how theatresports can be applied in educational settings as an alternative way to implement positive education and bring about positive outcomes for youth. Owing to the positive results, a practice implication is that the program can be launched in more tertiary educational settings and youth settings. Theatresports can be implemented in the form of interdisciplinary collaboration between theatrical practitioners, education practitioners, and helping professionals such as social workers and psychologists, combining the expertise of each profession to optimize the outcomes [46]. A prerequisite for such program implementation is to provide sufficient resources and adequate training for the professionals involved, so as to enhance their mutual understanding of the disciplines of one another and to facilitate their collaboration [47].

## Limitations of the study

This study was a cross-sectional study, which only captured participants' feelings and insights regarding the program specifically at a specific time, rather than investigating how the processes and components of the program contributed to the outcomes. Moreover, the age range of the participants was wide; this study only inquired into the outcomes of the program for the youth participants in general but not for those from different age groups.

## Future research

In the future, quantitative research could be conducted to reconfirm the qualitative outcomes of implementing theatresports as a form of positive education. Additionally, a longitudinal study could help to understand program participants’ involvement at different points in time and how this affects the program outcomes. Furthermore, such a study could be conducted for different age groups.

## Data Availability

The data that support the findings of this study are available on request from the corresponding author, Gloria Hongyee Chan. The data are not publicly available because they contain information that could compromise research participant consent.
